# Machine Learning based Model Reveals the Metabolites Involved in Coronary Artery Disease

**DOI:** 10.1177/11795972251352014

**Published:** 2025-07-08

**Authors:** Fathima Lamya, Muhammad Arif, Mahbuba Rahman, Abdul Rehman Zar Gul, Tanvir Alam

**Affiliations:** 1 College of Science and Engineering, Hamad Bin Khalifa University, Doha, Qatar; 2 Department of Biochemistry and Microbiology, North South University, Dhaka, Bangladesh; 3 Department of Biology, McMaster University, Hamilton, ON, Canada; 4 National Center for Cancer Care and Research, Hamad Medical Corporation, Doha, Qatar

**Keywords:** coronary artery disease, machine learning, metabolomics, metabolites, Qatar Biobank

## Abstract

**Introduction:**

Coronary artery disease (CAD) is a major global cause of morbidity and mortality. Therefore, advances in early identification and individualized treatment plans are crucial.

**Methods:**

This article presents machine learning (ML) based model that can recognize metabolomic compounds associated with CAD in the Qatari population for the early detection of CAD. We also identified statistically significant metabolic profiles and potential biomarkers using ML methods.

**Results:**

Among all ML models, artificial neural network (ANN) outstands all with an accuracy of 91.67%, recall of 80.0%, and specificity of 100%. The results show that 173 metabolites (*P* < .05) are significantly associated with CAD. Of these metabolites, the majority (95/173, 54.91%) were high in CAD patients, while 45.09% (78/173) were high in the control group. Two metabolites 2-hydroxyhippurate (salicylurate) and salicylate were notably higher in CAD patients compared to the control group. Conversely, 4 metabolites, cholate, 3-hydroxybutyrate (BHBA), 4-allyl catechol sulfate, and indolepropionate, showed relatively higher level in the control group.

**Conclusion:**

We believe our study will support in advancing personalized diagnosis plan for CAD patients by considering the metabolites involved in CAD.

## Introduction

Coronary artery disease (CAD) possess a huge burden on healthcare and is one of the major concerns worldwide. Atherosclerotic plaque buildup in the arterial lumen, commonly known as CAD, results in reduced blood flow and impairs the myocardium’s ability to receive oxygen.^1^ The 4 types of CAD include nonobstructive coronary atherosclerosis (NOCA), unstable angina pectoris (UA), acute myocardial infarction (AMI), and stable angina pectoris (SA). These categorization are based on the severity of the cardiac injury, clinical symptoms, and amount of arterial blockage. Additionally, acute coronary syndrome (ACS) is used to describe UA and AMI.^2^ The incidence and related mortality of CAD could be reduced if the molecular causes behind the condition could be understood. The development of CAD is characterized by several intricate molecular events. Angina and AMI are frequently caused by atherosclerosis, which is a gradual and intricate process.^3^ Early detection and differential diagnosis of CAD enable the initiation of the optimal patient-specific treatments. Based on the electrocardiogram (ECG), stress tests, cardiac markers, symptoms, and coronary angiography, the current clinical diagnosis distinguishes between the various forms of CAD.^3-6^ However, numerous studies have shown that incorporating new biomarkers such as coronary artery calcium, cardiac troponin-T, or a hereditary risk score, can improve the assessment of CHD risk.^7^ Nevertheless, these investigations have identified only a few unique and manageable biochemical processes. Other technologies, such as metabolomics, could be valuable in identifying new biomarkers and pathways connected to the development and progression of CAD.^7^

Metabolomics is a relatively recent field within the omics field originating from the prestigious discipline of analytical biochemistry. It is also closely related to other omics fields such as genomics, transcriptomics, and proteomics.^8^ Due to recent technological advancements, we can now focus on metabolite analysis in various biological specimens, which has become increasingly important for studying human illnesses like cardiovascular disease. Abnormal metabolism is another indication of CAD is abnormal metabolism.^8^ Advancement in OMICS-based technologies have made the analysis of CVD biomarkers more specific, rapid and efficient.^8,9^ By combining metabolomics techniques in conjunction with other technologies such as transcriptomics, proteomics, and genomics, we have recently gained a better understanding of the pathophysiologic mechanisms underlying CAD and cardiovascular disease (CVD), as well as identify biomarkers that may offer potential treatment strategies.^9,10^ Metabolomics has the potential to simultaneously identify a larger number of metabolites than standard laboratory approaches, facilitating a comprehensive understanding of biological events and metabolic pathways.^11^ The appeal of metabolic profiling lies in the fact that small percentage of endogenous human metabolites aproximately 7000—are associated with predictable genes (25 000), transcripts (100 000) and proteins (1 000 000).^12^ However, the human metabolome is far more complex, influenced by environmental factors such as pollution, gut microflora, and nutrition. Metabolomics provides a unique opportunity to investigate the interactions between genotype and environment, as well as genotype and phenotype, considering that the organism’s metabolome and genotype are closely linked. Metabolites exhibit significant chemical variation and are present in varying concentrations. As a result, it is not feasible to precisely detect every metabolite in human specimens in a single experiment using a single analytical tool.^8,12^

This article presents a significant contribution to metabolomics and coronary artery disease (CAD) detection in the Qatari population through the development and evaluation of an AI-powered model. The key findings of this study are as follows:

- We are introducing a comprehensive dataset of 116 anonymous samples with 1159 metabolite features collected from the Qatar Biobank (QBB) for the CAD cohort. The dataset included both CAD patients (54.31%) and control samples (45.69%), providing a balanced and diverse set of metabolite data along with demographic and clinical information.
- We propose 173 statistically significant metabolites for downstream analysis. The proposed ANN based machine learning classification models were able to detect CAD with high accuracy over 91%.
- We identified multiple metabolites that is, 2-hydroxyhippurate, salicylate, cholate, 3-hydroxybutyrate, 4-allyl catechol sulfate, and indolepropionate as being associated with CAD. Pathway analysis revealed galactose metabolism, starch and sugar metabolism, and sphingolipid metabolism pathway, which are crucial for cellular activity in CAD patients.

## Background Studies

Metabolomics is a promising approach for understanding the metabolic changes associated with the onset, progression, and prognosis of CAD. Studies using metabolomic profiling have revealed unique metabolic signatures or patterns linked to CAD, providing new insights into the molecular pathways involved in the disease’s pathogenesis study^13^ identified significant differences in phospholipid metabolites such as choline, glycerophosphoethanolamine, and glycerophosphorylcholine between OIS and OIR, as well as type 2 diabetes melitus (T2DM). Alterations in metabolic indicators like 3-hydroxymyristate, dimethylarginine, and 1,5-anhydroglucitol indicated vascular, liver, and type 2 diabetes issues, which were further validated. In comparison to healthy controls, another study^14^ found that babies born to women with polycystic ovary syndrome (PCOS) had lower birth weights. There was a negative correlation between birth weight and systolic blood pressure (SBP), while the PCOS group showed significantly higher triglyceride levels. Enrichment analysis revealed elevations in arachidonic acid, linoleic acid, and palmitic acid. The area under the curve (AUC) for predictors of low birth weight (2500 g) was 0.88, identifying several indicators related to infant birth weight. According to another study,^15^ vitamin D deficiency is more common in younger individuals, whereas dyslipidemia is more common in older people even when they get vitamin D levels. Phosphatidylcholine and phosphatidylethanolamine levels significantly change in case of vitamin D insufficiency without dyslipidemia, while sphingomyelins were more altered. Those with adequate levels of vitamin D had higher levels of ergothioneine. Dyslipidemia may affect the skin’s ability to synthesize vitamin D from 7-dehydrocholesterol.

The end products of gluconeogenesis, including glucose and its polymer, were found to be present in higher amounts in individuals with T2D, according to the study.^16^ Elevated levels of glutamate and glycine-betaine, 2 important Osmo protectants used by Salmonella typhimurium, were also detected. Some individuals showed abnormal levels of citrate, glutinane, and alanine, indicating possible paraquat poisoning, while others had salicylate (aspirin) detected in their urine and blood. The study suggested that people with diabetes should take aspirin to reduce their risk of heart attacks caused by coronary blockage. The study revealed multiomics correlations at CpG sites,^17^ encompassing smoking-specific metabolites, proteins, and diabetes markers. Mendelian randomization indicated that the methylation of obesity-associated CpG sites, such as DHCR24, MYO5C, and CPT1A, is causally influenced by metabolite levels. Another study^18^ explored the feasibility and limitations of using the Glycomark™ assay to measure 1,5-AG in saliva for diabetes screening. Although saliva serves as a stable matrix in biochemical experiments, the predominant signal in saliva is galactose, which is absent in blood but resembles 1,5-AG. The study found that individuals with insulin sensitivity (IS) exhibited lean/overweight characteristics, while those with insulin resistance (IR) had elevated levels of long-chain fatty acids and microbiota byproducts, such as the phenylalanine derivative carboxyethylphenylala-9. Additionally, androgenic steroids, such as androsterone glucuronide, were higher in IR participants.^19^ Another study^20^ highlighted 3 types of metabolites associated with glycemic control in plasma, urine, and biofluids. Urine metabolites were associated with 1 or 2 timeframes of glycemic regulation, while plasma metabolites were linked to all 3 timescales. Elevated levels of lactate in urine were specific to long-term glycemic control, whereas the upregulation of malate in urine was associated with acute dysregulation and short-term glycemic control. The case-control methodology may have identified metabolites linked to diabetes that are not directly affected by fluctuations in glucose homeostasis. These metabolites may include metformin, phenylalanine, isobutyrylcarnitine, cysteine, alanine, pipecolate, and various metabolites found in urine.^20^

Machine learning was applied in the study^21^ to understand the multivariate nature of blood pressure (BP) regulation. In the TwinsUK cohort, the model explained 39.2% of the variance in systolic blood pressure (SBP), and in an ethnically diverse Qatar Biobank (QBB) sample, 45.2% of the variance. According to this study, the metabolome, 5 nutrient intakes from food, and 7 biochemical markers were the key factors influencing blood pressure regulation. Among non-demographic factors total fats, trans fat, and saturated fat intake with dihomo-linolenate, cis-4-decenoyl carnitine, lactate, chloride, urate, and creatinine, had the most significant effect.^21^ Another study^22^ showed that, hypertensive patients—especially those with T2D and hypertension—who also have severe COVID-19 experiences altered lipid metabolism. Long-chain polyunsaturated fatty acids, particularly those with lower tri- acylglycerol levels, were identified as potential targets for diagnosis and treatment, indicating that lower triacylglycerol levels may worsen hypertension in these patients. In a separate study,^23^ serum from COVID-19 patients was examined to identify molecular markers that could predict clinical outcomes and shed light on the disease’s pathophysiology. The study found protein-metabolite relationships related to energy metabolism, collagen breakdown, vascular homeostasis, and immune regulation. Several proteins and metabolites were associated with clinical indicators linked to long-term mortality and morbidity. A composite outcome measure was developed, which predicted severe disease with a concordance score of about 0.69 and strong predictive power (0.83-0.93) across 2 different datasets.^23^ One research study^24^ in the Middle East uncovered new and established metabolic pathways and circulating compounds related to coronary heart disease (CHD). Enriched pathways included sphingolipid metabolism, glycolysis, the oxidation and breakdown of branched-chain fatty acids, and the metabolism of D-arginine and D-ornithine. Magnetic resonance spec- troscopy (MRS) was highlighted as a diagnostic and predictive tool due to its strong discriminative ability between controls and CHD patients. We have summarized the results from the above-mentioned articles in Table 1.

**Table 1.** Summary outcome from the previous studies focused on metabolomics of CAD patients in Qatar.

Study	Year	Cohort size	Disease	Metabolites	ML used	Outcome
Al-Sulaiti et al^13^	2019	107	Diabetes	OIS: choline, GPC, 1,5-AG or their comorbidities OIR: Glycerophosphoethanolamine, glucose.	No	N/A
Diboun et al^14^	2021	68	PCOS pregnant women	Triglycerides containing arachidonic acid, palmitic acid, linoleic acid, hexosylceramide, ceramide, and serine.	No	N/A
Mousa et al^15^	2022	227	Vitamin-D deficient people	Sphingomyelins, PC, PE, N-stearoyl-sphingosine (ceramide), lysophospholipids.	No	N/A
Ullah et al^16^	2015	348	Diabetes	Dimethylamine, betaine, mannitol, N,Ndimethylglycine, glucose, and b-alanine	No	N/A
Zaghlool et al^17^	2018	359	Diabetes	1,5-AG,glucose, o-cresol sulfate (colorectal cancer)	No	N/A
Halama et al^18^	2016	82	Diabetes	1,5-AG,galactose	No	N/A
Diboun et al^19^	2020	200	Lean/overweight IR females	1-Carboxyethyl phenylalanine, gamma-Glutamyl phenylalanine, 10-heptadecenoate, fructosyllysine, N-acetyl = aspartyl-glutamate, N-acetylglycine, eicosoneote, glucoronide, palimitoleate.	No	N/A
Yousri et al^20^	2015	369	Diabetes	1,5-AG,2-hydroxybutyrate, b-hydroxypyruvate, 4-hydroxyphenylpyruvate, xylonate, gluconate, ribose, mannose.	No	N/A
Louca et al^21^	2022	2807	Blood pressure	Urate, dihomo-linolenate, lactate, Cis-4-decenoyl carnitine	XGBoost	MAE = 11.342 ± 0.348
Elrayess et al^22^	2022	115	Covid-19 (Diabetes and BP patients)	Decreased levels of triglycerides (palmitic acid, oleic, docosahexaenoic acid, docosapentaenoic acid.	No	N/A
Buyukozkan et al^23^	2022	427	Diabetes	Phenylalanine, tryptophan, sphingolipids, hexanoylcarnitine, L-carnitine, caprylic acid, cytosine, arachidonic acid.	LASSO Regression	Concordance index 0.69

## Materials and Methodology

**Figure 1.**
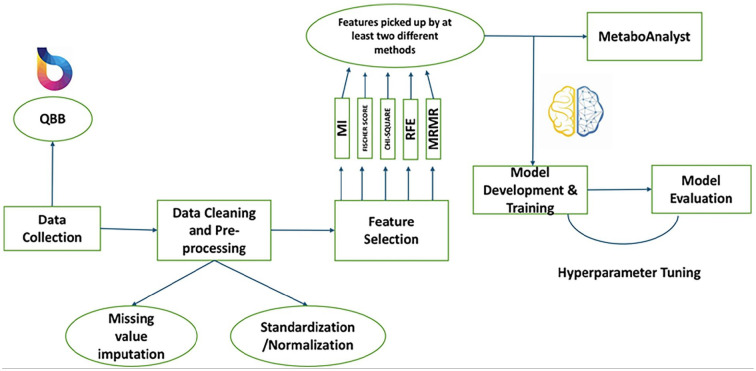
Workflow diagram of the steps considered for this study.

Upon receiving the IRB approval, we first collected the biomedical records for a pool of participants from QBB. Then we cleaned the dataset applying data pre-processing steps. Then we extract biomedical features form the dataset of the participants. Finally, machine learning models were used to separate the CAD group from the control group with important features. Figure 1 summarizes the key steps of the workflow of this study.

### Data Collection from Qatar Biobank (QBB)

Collecting a valid benchmark dataset is an essential phase for training and validating a machine learning model.^25,26^ Herein, a dataset of 116 anonymous samples and their metabolite features were collected from the Qatar Biobank (QBB) for the coronary artery disease (CAD) cohort. The dataset had 53 controls (45.69%) who were free from CAD) and 63 patients (54.31%), which consisted of heart attack, heart revascularization, stroke, angina and more than 1 case.

### Data Description

The dataset consists of sample types, and their 1159 metabolites from blood, along with other morphological information such as age, BMI, gender and other key information of participants.

### Data Cleaning and Pre-processing

Developing an efficient predictor passes through series of steps.^27^ Data cleaning is considered a key pre-processing method that helps to enhance the proposed model performance. We used various pre-processing algorithms in Jupyter Notebook IDE with Python 3.6 for removing the noise, irrelevant attributes, and redundancy from the raw data samples.^28^ Initially, the data consisted of features (metabolites) with many values that needed to be filled in. Features with a high number of missing values were dropped, leading to the exclusion of 30 metabolites. In the next step, metabolites with more than 20% missing values were removed, leading to the exclusion of 344 metabolites. For the remaining metabolites, missing value imputation was performed by the *k*-nearest neighbor (KNN) imputer with *k* = 3. To identify statistically significant features from the remaining 785 metabolites, we applied 4 feature selection techniques, including, (a) Fisher’s score, (b) chi-square, (c) Recursive Feature Elimination (RFE), and (d) Minimum Redundancy Maximum Relevance (mRmR) were performed. If a feature was selected by at least 2 of the aforementioned methods, we considered that feature for downstream analysis. Following this approach, we selected 53 statistically significant metabolites.

### Feature Scaling Techniques for Metabolites

Scaling techniques such as standardization and normalization (min-max scaling) were performed separately on metabolites to verify which works best for the machine learning model.

(1) $$\mathrm{Standardization}\;\mathrm{formula}:\;x_{\mathrm{standardized}}\;=\;\frac{x-\mu }{\sigma }$$

(2) $$\mathrm{Min}-\mathrm{MaxScaling}:x^{\prime }=\frac{x-\min\left(x\right)}{\max\left(x\right)-\min\left(x\right)}$$

(3) $$Accuracy=\frac{TP+TN}{TP\;+\;TN\;+\;FP\;+\;FN}$$

### Statistical Analysis of the Features

Statistical analysis of the features was conducted using the software “JASP.” The software provided information such as the mean of metabolites for both the control and CAD patients as well as standard deviation for each group. “Student *T*-test” and “Mann-Whitney” tests were applied to calculate the *p*-values for each metabolite, which helped to determine the significant and non-significant metabolites.

### Machine Learning Modeling

Multiple machine learning classification models such as Logistic Regression (LR), Support Vector Machine (SVM), Naïve Bayes (NB), Decision Tree (DT), Random Forest (RF), XGBoost (XGB), CatBoost (CB), and Artificial Neural Network (ANN) were used to determine control class = 0 and CAD class = 1. We optimized the hyperparameters of the models using GridSearchCV of the Python library.

### Model Evaluation

A fivefold cross-validation technique was employed to evaluate the model performance, in which the data is split into 80% training and 20% validation data.^29^ The model’s performance metrics were calculated for each fold, and the average was taken for the result. Performance metrics used for evaluation were Accuracy, Precision, Sensitivity, Specificity, and *F*1-score as in our previous study.^30^ Area under the curve (AUC) of receiver operating characteristics (ROC) cure was also calculated for model evaluation.

(4) $$Precision=\frac{TP}{TP+FP}$$

(5) $$Sensitivity=\frac{TP}{TP+FN}$$

(6) $$Specificity\;=\frac{TN}{\;FP\;+\;TN}$$

(7) $$F1\;=\;\frac{2\;\times \;\left(precision\;\times \;sensitivity\right)\;}{precision\;+\;sensitivity}$$

where *TP*, *FN*, *FP*, and *TN* stand for True Positive, False Negative, False Positive, and True Negative, respectively.

## Results and Discussion

### The Baseline Characteristics of the Cohort

There were a total of 116 samples overall, consisting of 53 controls (45.69%) were free from CAD and 63 patients (54.31%), which included heart attack, heart revascularization, stroke, angina and multiple cases. Out of 116 participants, 43 (37.07%) female, and 73 (62.93%) were male. Every participant in this study is from the Qatari population- either Qatari nationals or long-term residents of Qatar.

### Result of Statistical Analysis

Of 785 metabolites, 173 were statistically significant (*P* < .05) between the control and CAD. The summarized metabolites and their *P*-values are provided in Supplemental Table S1. The majority of metabolites (95/173, 54.91%) were higher in CAD, whereas 45.09% (78/173) metabolites seemed to be elevated in the control group.

**Figure 2.**
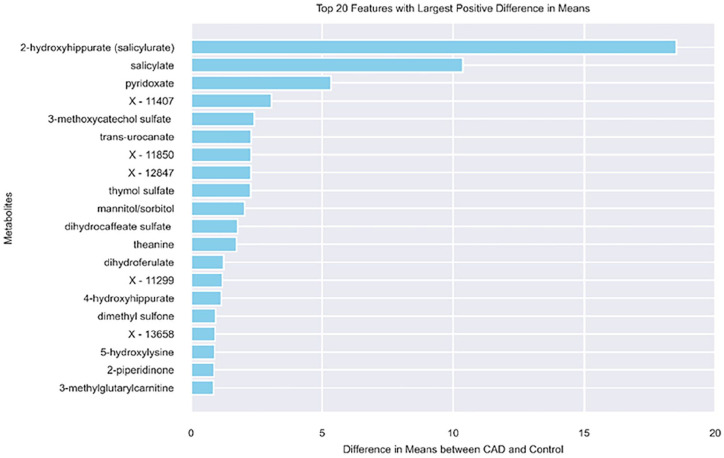
Top 20 metabolites with the largest difference in the mean between CAD and control.

Figure 2 shows the top 20 metabolites with largest difference between CAD and control (higher value in CAD compared to control). For 2 metabolites 2-hydroxyhippurate (salicylurate) and salicylate we observed a very high value in CAD compared to the control group. 2-Hydroxyhippurate, or salicyurate, is an aryl glycine conjugate that the body produces to get rid of too many salicylates, such as aspirin. According to the study,^31^ itwas strongly linked to a 20% increased risk of type 2 diabetes, but Fasting Glucose or 2 hour Glucose levels, insulin secretion, or insulin sensitivity were not significantly affected by this metabolite. Higher hippurate levels have been associated with more diverse gut flora, according to the study,^32^ which raises the possibility that 2-hydroxyhippurate is a marker of the abundance of gut bacteria. Hippurate levels were favorably correlated with fruit and whole grain consumption. Hippurate was found to be trending upward and was linked to a lower risk of metabolic syndrome (MetS), indicating the importance of gut microbiota in diet-related effects on metabolic health.^32^ Hippurate and Salicylate metabolites are part of Aspirin medicine. Probably, the CAD patients are taking Aspirin as a medication. Bioactive chemical molecules known as salicylates are found naturally in dietary products. Acetylsalicylic acid is the most often used derivative of salicylic acid. Natural and artificial salicylates are beneficial to human health in several ways. Their anti-inflammatory characteristics aid in the treatment of inflammatory illnesses. Some may guard against neurological disorders, while others may prevent cancer.^33^ Additionally, several salicylates may aid in the management of diabetes. When taken consistently, it also exhibits anticoagulant qualities.^34-36^ According to the study,^37^ Salicylic acid concentrations in serum samples were higher in vegetarians than non-vegetarians. Salicylic acid concentrations were also noticeably greater in those on a low dose of aspirin. Nevertheless, some persons may experience hypersensitivity reactions if aspirin or acetylsalicylic acid are taken with other nonsteroidal anti-inflammatory drugs (NSAIDs) as aspirin’s widespread use as an anti-inflammatory drug, this hypersensitivity is often referred to as aspirin hypersensitivity.^38^ This study also found that a customized low-salicylate diet effectively alleviated asthma, rhinosinusitis, and urticaria symptoms in patients hypersensitive to salicylates. People with irritable bowel syndrome (IBS) did not significantly differ in their symptoms, according to a study.^39^ Nevertheless, 2 individuals—1 of whom was aspirin-sensitive, and the other was exhibiting worsening symptoms—exhibited apparent symptom provocation following a high-salicylate meal, indicating that salicylates found in food may influence the onset of symptoms in certain IBS patients. Another study^40^ reveals that salicylate, an aspirin metabolite, non-competitively reduces the maximal current produced by native glycine receptors, mainly focusing on receptors that contain the *α*1 subunit. When the *α*1-subunit is mutated, salicylate inhibition is removed, making receptors open to negative consequences.

**Figure 3.**
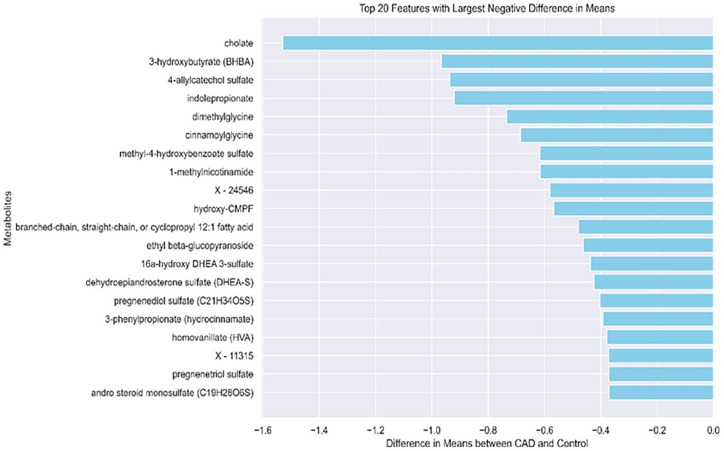
Top 20 metabolites with the lowest difference in the mean between CAD and control.

On the other hand, we observed relatively higher control values for 4 metabolites, cholate, 3-hydroxybutyrate (BHBA), 4-allyl catechol sulfate, and indolepropionate relative to the CAD. Figure 3 shows the top 20 metabolites with largest difference between control and CAD (higher value in control compared to CAD).

One essential bile acid that is produced in the liver from cholesterol is called cholic acid. Together with chenodeoxycholic acid, it is one of the 2 primary bile acids that the liver produces. The rate-limiting step in the manufacture of bile acid, cholesterol-7-*α*-hydroxylase, is downregulated by cholic acid.^41^ When coupled with amino acids such as taurine or glycine, cholate, the conjugate form of cholic acid, generates a bile salt that helps to solubilize dietary lipids in the intestines and form micelles for improved absorption.^42^ As a hormone affecting the liver and extrahepatic tissues, cholic acid facilitates the intestinal absorption of hydrophobic substances. It modifies the actions of receptors through interactions. Variations in bile acid content can cause extrahepatic diseases; however, interpreting these changes can be difficult due to their complexity.^43,44^ Impaired cholate transport and homeostasis resulting from cholestatic disorders can cause gastrointestinal disorders such as irritable bowel syndrome, inflammatory bowel disease, and cancers of the colon, stomach, and esophagus, as well as liver diseases such as nonalcoholic fatty liver disease, hepatocellular carcinoma, and cholangiocellular carcinoma. The study^45^ investigates the utilization of cholic acid supplementation in patients with autosomal recessive PEX gene mutations who have Zellweger spectrum disorders. The results show reduced plasma bile acid intermediate levels, bile acid synthesis, and urine excretion. When blood glucose levels are low, animals use 3-Hydroxybutyrate (3-HB) as a backup energy source created during fatty acid oxidation.^46^ Up to 60% of the energy used by the human brain during extended fasting comes from 3-hydroxybutyrate (3HB), a crucial ketone substance. Both human milk and adipose tissue contain it. Brain disorders such as multiple sclerosis, epilepsy, stroke, Parkinson’s, Alzheimer’s, Huntington’s, depression, and schizophrenia can all be impacted by abnormal levels.^47^ It also controls metabolic rate, neural activity, lipid metabolism, and gene expression. Histone deacetylases are inhibited by epigenetic regulation, which may have an impact on conditions like depression, cancer, and neurological illnesses.^48^ Blood-brain, intestinal, and skin barrier functions are all impacted by BHB, which may impact permeability, immunological responses, gastrointestinal health, inflammation, and wound healing.^49^ According to a study,^50^ Indolepropionic acid (IPA), a product of the gut microbiota, is inversely linked to type 2 diabetes risk as well as low-grade inflammation in high-risk patients. Insulin secretion was strongly connected with dietary fiber intake, while IPA levels were directly linked to insulin secretion. IPA lowers endotoxin levels, suppresses NF-*κ*B, preserves intestinal homeostasis, controls gut microbiota, and protects the liver.^51^ IPA is essential for preserving energy homeostasis and averting oxidative stress damage by suppressing the production of proinflammatory cytokines. IPA synthesis, however, can be decreased by atherosclerosis risk factors.^52^ IPA efficiently targets mycobacteria, including ones resistant to drugs, by focusing on amino acid production. It can be found in both healthy people and TB patients, and because of its antioxidant and anti- inflammatory qualities, it can be used to treat diseases related to tuberculosis.^53^ It also increases the function of the gut barrier by inducing the expression of tight junction proteins, which keep harmful substances out of the gut. By regulating blood flow to the organs, it can preserve systemic homeostasis and possibly identify and treat conditions, including breast cancer, Alzheimer’s disease, and non-alcoholic fatty liver disease (NAFLD).^54^ After the feature selection, we used the following metabolites as the important features highlighted in Supplemental Table S1.

### Machine Learning Models Performance

Different machine learning models and their performance metrics are shown here in Table 2 for the validation set. Performance metrics of the ML models on the validation set are given in Table 2. Overall, ANN outstands all the ML models with an accuracy of 0.9167, precision-1.00, recall-0.800, specificity-1.00, *F*1 score of 0.8889, and MCC of 0.8367. SVM with MinMax Scaling had remarkable validation set performance with an accuracy of 82.79%, precision of 84.14%, and specificity of 84.55%. This model’s MCC value of 0.669 shows that it performs better in binary classification jobs. With accuracy rates of 81.05% and 80.18%, the Logistic Regression models demonstrated excellent consistency while keeping recall and precision in check. Additionally, random forest models performed well, with 78.48% accuracy achieved with standard scaling in particular. The MinMax Scaling method did, however, result in a minor improvement in accuracy to 79.31%, indicating that its performance is relatively resilient to the scaling technique used. With the lowest validation accuracy of 56.88%, Decision Tree models demonstrated the most significant fluctuation in performance, suggesting a greater propensity for overfitting and a less successful capacity for generalization on the validation data. Despite having flawless training scores, the XGBoost and Naive Bayes models had lower validation accuracies of roughly 77.57% and 71.59%, respectively. This suggests there may be overfitting problems and difficulties in balancing model complexity and generalization ability. Calibration curve and Brier score for the prom-sing models are presented in Supplemental Figure S1.

**Table 2.** Summary of ML model results for the validation set.

Model	Normalization	Accuracy	Precision	Recall	*F*1 score	Specificity	MCC	AUC
AUC	(Standard Scaling)	0.810	0.828	0.808	0.805	0.789	0.636	0.65
SVM	(Standard Scaling)	0.802	0.810	0.805	0.800	0.847	0.615	0.57
NB	(Standard Scaling)	0.715	0.731	0.721	0.712	0.807	0.452	0.53
RF	(Standard Scaling)	0.784	0.800	0.782	0.779	0.752	0.582	0.62
XGB	(Standard Scaling)	0.775	0.795	0.775	0.771	0.770	0.570	0.72
CB	(Standard Scaling)	0.792	0.814	0.786	0.785	0.730	0.599	0.74
DT	(Standard Scaling)	0.594	0.609	0.593	0.581	0.598	0.202	0.15
ANN	(Standard Scaling)	0.916	1.00	0.80	0.888	1.00	0.836	0.86
LR	(MinMax Scaling)	0.801	0.818	0.800	0.797	0.789	0.618	0.60
SVM	(MinMax Scaling)	0.827	0.841	0.828	0.825	0.845	0.669	0.55
NB	(MinMax Scaling)	0.715	0.731	0.721	0.712	0.807	0.452	0.53
RF	(MinMax Scaling)	0.793	0.800	0.790	0.787	0.769	0.590	0.63
XGB	(MinMax Scaling)	0.775	0.795	0.775	0.771	0.770	0.570	0.73
CB	(MinMax Scaling)	0.792	0.814	0.786	0.785	0.730	0.599	0.74
DT	(MinMax Scaling)	0.568	0.581	0.568	0.555	0.563	0.149	0.20
ANN	(MinMax Scaling)	0.875	0.888	0.80	0.842	0.928	0.741	0.80

### Comparison of ML Model Against Pre-test Calculator for CAD

To compare our prediction performance against clinical standard pre-test CAD calculator,^55^ we used the online version of Pre-test calculator from CAD Consortium https://qxmd.com/calculate/calculator_287/pre-test-probability-of-cad-cad-consortium. We tested all the CAD patients in this calculator. Among all the CAD patients of our cohort, the “Clinical Model” of Pre-test calculator were able to predict only 8 out of 63 CAD patients indicating just over 12% accuracy. On the other hand, out proposed ML model were able to detect 80% of CAD patients correctly (See Table 2). This warrants the need for inclusion of additional biomarkers in CAD calculator which may improve the early detection of CAD patients.

### Comparison of Our Findings with Previous Works on CAD-Related Metabolites

There are several metabolites from our findings which intersect with other literature. Metabolites involved in bile acid metabolism, such as Cholate and taurocholenate sulfate, are highly likely to be considered potential metabolites in CAD. Studies^56-58^ reveal reduced levels of circulating bile acid in patients who have coronary artery disease (CAD), and glycochenodeoxycholic acid (GCDCA) is a major predictor and marker of CAD. Furthermore, compared to non-CAD patients, CAD patients had lower amounts of bile acid excretion. Bile acids have therapeutic potential for metabolic disorders due to their critical role in lipid metabolism, inflammation, and signaling. Atherosclerosis, arrhythmia, myocardial infarction, diabetes cardiomyopathy, and heart failure are just a few of the cardiovascular disorders that are linked to abnormal bile acid metabolic pathways. These pathways, which function as ligands for different receptors and regulate metabolism, have also been linked to cardiac dysfunction. One new approach for possible CVD treatments could be to modify BA signal transduction by synthesis and composition. Bile acids are promising targets for treating metabolic disorders because of their essential roles in inflammation, signaling, and lipid metabolism. The metabolite eicosenedioate (C20:1-DC), which is involved in fatty acid and dicarboxylate metabolism seems to be highly associated with CAD. The 20-carbon fatty acid eicosanoic acid serves as a building block for lipid compounds known as eicosanoids, which are implicated in biological processes such as inflammation. An additional factor in atherosclerosis and CAD is dysregulation of lipid metabolism. The development of atherosclerosis is aided by eicosanoids that regulate inflammation, vascular tone, platelet aggregation, and endothelial function. Eicosanoid production imbalances can worsen plaque accumulation, constrict coronary arteries, and raise the risk of CAD.^59,60^ The metabolites which are involved in Leucine, Isoleucine and Valine Metabolism (Amino Acid super pathway) such as N-methylpipecolate, 5-hydroxylysine, N, N,N-trimethyl-5-aminovalerate and Branched Chain Amino Acids (BCAA) are strongly correlated with CAD. Elevated levels of BCAA, associated with metabolic problems, could exacerbate cardiovascular disease by activating mTOR and causing mitochondrial dysfunction.^61^ The study^62^ discovered that Branched chain amino acids, linoleic acid, and Larginine are significant metabolites linked to T2DM-associated CAD and may have predictive value. According to many studies^63-68^, Arginine metabolism has an impact on CAD, a vascular disease that can accelerate the disease’s progression by impairing nitric oxide generation and increasing vasoconstriction, inflammation, and thrombosis, but it is not present in our significant metabolites.

In addition, we observed that acylcarnitines such as stearoylcarnitine and methyl-succinoylcarnitine are metabolites closely linked to CAD. Based on the literature^69,70^ there is a significant correlation between coronary artery disease (CAD) and specific acylcarnitines, including oleoylcarnitine, linoleylcarnitine, palmitoyl carnitine, and stearoylcarnitine. This suggests that elevated serum acylcarnitine levels may help with patient stratification and highlight the potential role of long chain acylcarnitines in cardiovascular mortality. The study^71^ finds that in individuals with non-obstructive CAD disease (NOCAD), higher serum, even chained acylcarnitines, especially palmitoylcarnitine, can predict a poor long-term prognosis. Metabolites Which are involved in sphingolipid metabolism such as sphingomyelin (d18:1/24:1, d18:2/24:0), palmitoyl sphingomyelin and hydroxypalmitoyl sphingomyelin (d18:1/16:0(OH)), and the ceramide glycosyl-N-palmitoyl-sphingosine (d18:1/16:0) are observed to be significant biomarkers of CAD. Elevation in plasma sphingomyelin (SM) has been associated with a decreased left ventricular sys- tolic function and an increased risk of cardiovascular disease, which can result in heart failure and coronary heart disease.^72^ SM levels may be a sign of atherogenic residual lipoprotein buildup and have a positive correlation with coronary artery disease (CAD). Both the SM/PC (phosphatidylcholine) ratio and SM levels are higher in CAD patients.^73^ The studies^74-78^ investigate the function of sphingomyelins and ceramides in endothelial cells and how they affect coronary artery disease (CAD). Ceramides contribute to vascular dysfunction and CAD by impairing EC-dependent vasorelaxation, aggravating vasoconstric- tion, and reducing nitric oxide generation. Additionally, they have an impact on eNOS activity, which may have an effect on vascular health and result in CAD. Ceramides play a significant role in CAD, a disorder marked by atherosclerotic plaque instability and mortality. Ceramides build up in plaques and trigger lipoprotein aggregation. Ceramide-lowering tactics, such as blocking ceramide production enzymes or focusing on particular pathways, have demonstrated promise in the treatment of CAD.^79-81^ We observed in our study that Phospholipids and Glycerophospholipids such as 1-(1-enyl-palmitoyl)-2-linoleoyl-GPE (P-16:0/18:2), 1-(1-enyl-palmitoyl)-2-palmitoyl-GPC (P-16:0/16:0), 1-(1-enyl-palmitoyl)-2-linoleoyl-GPC (P-16:0/18:2), 1-stearoyl-2-linoleoyl-GPC (18:0/18:2), 1-(1-enyl-palmitoyl)-2-oleoyl- GPC (P-16:0/18:1), 1-(1-enyl-palmitoyl)-2-palmitoleoyl-GPC (P-16:0/16:1), and 1-stearoyl-2-linoleoyl-GPI (18:0/18:2) are also biomarkers toward CAD. Literature^58,59,82^ shows that patients with coronary artery disease (CAD) have been related to inflammation in relation to glycerophospholipids, specifically phosphatidyl-choline and phosphatidylethanolamine. Elevations in specific glycerophospholipids may offer protection against inflammatory processes. Patients with CAD experience inflammation and atherosclerosis due to dysregulation of glycerophospholipid metabolism. Metabolites linked to glycerophospholipids, such as *β*-pseudouridine, are implicated in the course of CAD and serve as biomarkers for the risk and prognosis of the condition. According to the study,^83^ The progression of coronary artery disease is highly linked to lipid species and metabolites such as cysteine, methionine, and glycerophospholipid metabolism. Different PC species affect the evolution of CAD, and PC-containing modules are associated with myocardial infarction and HDLC. Atherosclerotic coronary heart disease patients may benefit from taking ceramides and phosphatidylcholines together to predict cardiovascular events, according to a study.^84,85^ To forecast cardiovascular death, this combination generates a risk score. In conjunction with high-sensitivity troponin T, the score’s predictive power increases. Another category of Metabolites related to CAD involves fructose, mannose, and galactose metabolism, that is, Fructose and Mannose. According to the studies^86-88^ Implications for CVD include the possible relationship between mannose metabolism, galactose metabolism, and high fructose consumption. Because fructose is mainly processed in the liver, it raises the risk of cardiovascular diseases (CVDs) and can cause insulin resistance, dyslipidemia, and nonalcoholic fatty liver disease (NAFLD). CVD issues may also result from galactosemia, a hereditary condition that affects the metabolism of galactose. In patients with coronary artery disease and diabetes mellitus, metabolic biomarkers such as apolipoprotein B and acylcarnitine ratio, in conjunction with patient factors such as age over 65, history of heart failure, and acute coronary syndrome, independently predict severe adverse cardiovascular events.^89^ A study^90^ that looked at several metabolites in diabetic patients revealed that thymine, pelargonic acid, glucosamine: galactosamine, creatine, 3-hydroxybutyric acid, 2-aminoisobutyric acid, and hypoxanthine were all significantly linked to the onset of coronary artery disease (CAD). These findings indicate potential biomarkers for early detection. Apart from this, our significant metabolites include metabolites involved in Vitamin A metabolism, that is, Retinol and the metabolite pregnanediol sulfate, which comes under the category of pregnenolone steroids. Based on other studies,^91-93^ cardiovascular issues have been related to vitamin A deficiency and supplementation; severe deficits are associated with higher risk. Research employing Mendelian randomization and clinical trials has failed to demonstrate that vitamin A therapy significantly reduces the risk of cardiovascular events. Except for an increased risk of heart failure, cardiovascular issues are uncommon in people with rickets and osteomalacia. Regarding pregnanediol sulfate, Based on the literature,^94^ Pregnenolone pathway metabolites, such as pregnanediol sulfate and pregnanediol sulfate, were found to be generally inversely related to subsequent events of cardiovascular disease, indicating a potential protective role against CVD and calling for more research into the relationship between steroid metabolism and cardiovascular health.

## Conclusion

Our study discovers key metabolites linked to CAD in the Qatari population by utilizing targeted metabolomics in conjunction with an AI-powered model. The novel biomarkers we discovered through our study are Retinol, Stachydrine, gamma- glutamylisoleucine, 1-ribosyl-imidazoleacetate, mannonate, 2-aminoheptanoate, and octadecadienedioate (C18:2-DC). We propose metabolites involved in Bile Acid metabolism and Carbohydrate Metabolism, Pregnenediol sulfate, eicosenedioate, several phospholipid metabolites such as GPC and GPE and their variations, BCAAs and other Amino Acid Metabolites, Acylcarnitines, sphingolipids metabolites as a potential biomarker for the detection of CAD. By employing MetaboAnalyst 6.0, we have identified important metabolites related to many superpathways, such as lipids, amino acids, carbohydrates, peptides, Xenobiotics, and Nucleotides. This all-encompassing approach not only advances our knowledge of the metabolic foundations of CAD but also opens the door to novel therapeutic and diagnostic approaches catered to the distinct metabolic profile of the Qatari population. Further studies are required to validate these metabolites in larger cohorts and clinical setups. Our study contains some limitations that need to be highlighted. We have a small cohort of size 116 Qatari population, and out of these, only 63 were CAD patients. Therefore, the statistical analysis we conducted needs to be rigorously researched based on the availability of a larger cohort. In the future, we will extend our study using these tools.

To translate these findings into clinical practice, we first need to validate the metabolites we identified and ANN model we proposed in larger, multi-ethnic cohorts to ensure generalizability of ML model. Then, we can design a targeted metabolomic panel focusing on key biomarkers like 2-hydroxyhippurate, cholate, and optimized for high-throughput clinical testing while securing regulatory approvals and standards. After the evaluation of this panel, the final panel can be integrated with existing risk scores (eg, ASCVD) and embedded into electronic health record system to enhance CAD risk stratification. These facilities can be deployed in preventive cardiology for early screening of high-risk groups, enabling personalized interventions such as dietary adjustments or pharmacotherapy guided by metabolic profiles. Clinician education for the medical practitioners and AI-powered decision support tools will facilitate adoption, while health economic analyses and payer partnerships with industry can ensure long-term sustainability of this plan.

## Supplemental Material

sj-docx-1-bec-10.1177_11795972251352014 – Supplemental material for Machine Learning based Model Reveals the Metabolites Involved in Coronary Artery Disease Supplemental material, sj-docx-1-bec-10.1177_11795972251352014 for Machine Learning based Model Reveals the Metabolites Involved in Coronary Artery Disease by Fathima Lamya, Muhammad Arif, Mahbuba Rahman, Abdul Rehman Zar Gul and Tanvir Alam in Biomedical Engineering and Computational Biology

sj-xlsx-2-bec-10.1177_11795972251352014 – Supplemental material for Machine Learning based Model Reveals the Metabolites Involved in Coronary Artery Disease Supplemental material, sj-xlsx-2-bec-10.1177_11795972251352014 for Machine Learning based Model Reveals the Metabolites Involved in Coronary Artery Disease by Fathima Lamya, Muhammad Arif, Mahbuba Rahman, Abdul Rehman Zar Gul and Tanvir Alam in Biomedical Engineering and Computational Biology
