# Supplementary material for: Machine Learning based Model Reveals the Metabolites Involved in Coronary Artery Disease
Source: Biomed Eng Comput Biol. 2025 Jul 8;16:11795972251352014. doi: 10.1177/11795972251352014 (PMC12246536; doi:10.1177/11795972251352014)
Supplement: sj-docx-1-bec-10.1177_11795972251352014 – Supplemental material for Machine Learning based Model Reveals the Metabolites Involved in Coronary Artery Disease [file sj-docx-1-bec-10.1177_11795972251352014.docx]

**Figure S1:** Calibration Curve and Brier Score for promising models

| **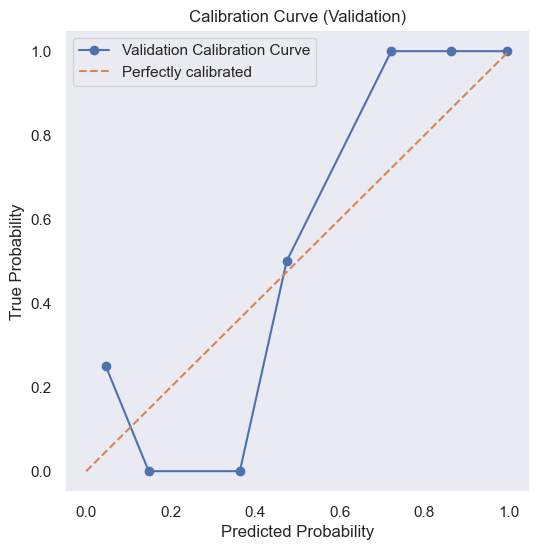** | **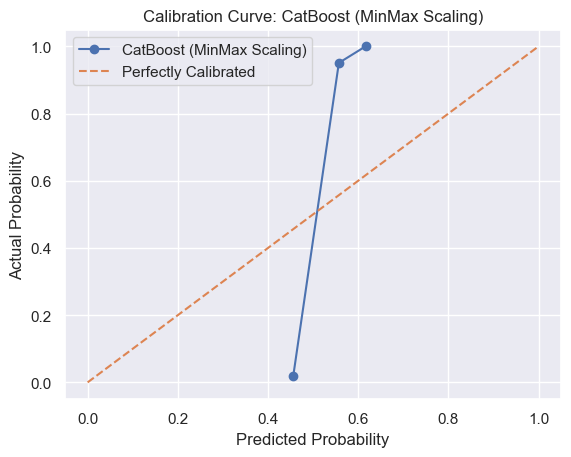** |
| --- | --- |
| **ANN (Brier Score: 0.112 )** | **CatBoost (Brier Score: 0.255 )** |
| 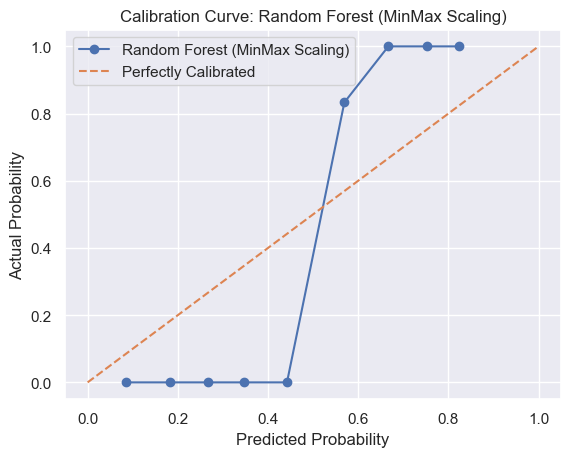 | 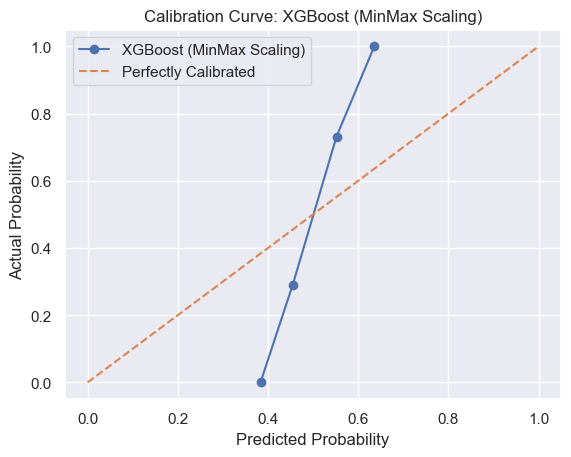 |
| **Random Forest (Brier Score: 0.232 )** | **XGBoost (Brier Score: 0.200)** |
| 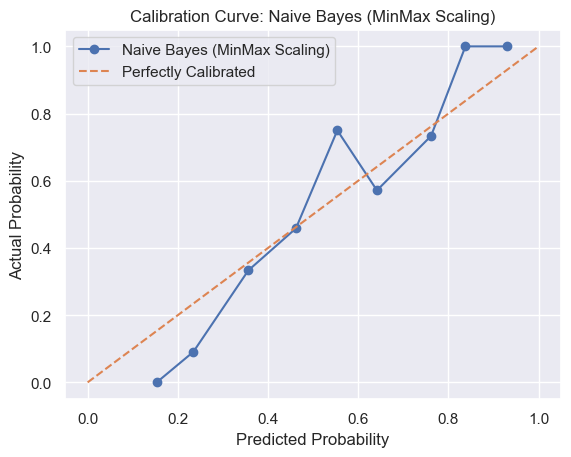 |  |
| **Naïve Bayes (Brier Score: 0.62 )** |  |
